# Editorial: In celebration of women in science: RNA networks and biology

**DOI:** 10.3389/fmolb.2023.1162378

**Published:** 2023-02-21

**Authors:** Anna M. Piccinini, Sybille Krauß

**Affiliations:** ^1^ School of Pharmacy, University of Nottingham, Nottingham, United Kingdom; ^2^ School of Science and Technology, Institute of Biology, University of Siegen, Siegen, Germany

**Keywords:** women in science, RNA, miRNA-microRNA, extracellular vesicles, exogenous miRNAs, myotonic dystrophy type 1, osteoarthritis, CXG repeat disorders

To date women are still underrepresented in disciplines like mathematics, computer science, natural sciences and technology, despite numerous efforts to reduce the gender gap in the field (Huang et al., 2020). Moreover, their visibility in terms of authorship and credits for their scientific achievements is still behind men (West et al., 2013; Ross et al., 2022). The field of RNA biology is no exception to this with notorious examples including Louise Chow and Sara Lavi, two of the six women who discovered RNA splicing. Despite being first authors of the papers reporting the discovery and independent scientists with their own research grants, Chow and Lavi were overlooked for the 1993 Nobel Prize and were not invited to or acknowledged during the celebrations of the 20th and 40th anniversaries of the discovery of RNA splicing in 1997 and 2017, respectively, at the Cold Spring Harbor Laboratory (CSHL) in New York (Geraldine, 2020). With this Research Topic we promote the work of women scientists on RNA Networks and Biology and help increase their visibility. We especially encouraged early career researchers to team up with senior female colleagues, which is one measure to promote a collaborative network that should positively impact the publication records and careers of women scientists. This Research Topic, co-authored by 39 researchers in the field, achieved its goal to promote authorship of women in visible positions (first author and last author). 4 out of 5 articles (80%) are led by a female first author, and female last authors are found in 4 out of 5 articles (80%).

The papers published in this Research Topic highlight diverse areas of RNA research in which women play a central role. Three original articles describe experimental work on transcriptome changes in myotonic dystrophy type 1 (DM1), the role of extracellular vesicle miRNAs and tRNAs in synovial fibroblast senescence, and the effects of *Moringa oleifera* miRNAs on lipid metabolism. Furthermore, one methods article reports advances in endogenous RNA pull-down. Finally, the Research Topic culminates in a review on intracellular and extracellular transport of RNA organelles in physiological conditions and neurodegenerative diseases.

Nearly two decades ago it was discovered that RNA is not only found in the nucleus and specialized regions of the cytoplasm of living cells, but also in extracellular vesicles (EVs) that are released by many cell types into the extracellular space (Ratajczak et al., 2006; Valadi et al., 2007). Various types of RNA molecules have been identified in EVs including small non-coding RNAs (sncRNAs), fragmented and intact mRNA, ribosomal RNA (rRNA) and long non-coding RNAs (lncRNAs). Notably, EV RNA can be taken up by specific cells and impact their gene expression and function (O'Brien et al., 2020). In this Research Topic, Wijesinghe et al. combine transcriptomic and proteomic approaches with computational analysis to identify differentially expressed sncRNAs and differentially abundant proteins in senescent EVs compared to non-senescent EVs in the context of osteoarthritis (OA). Several differentially expressed miRNAs and tRNAs are identified in EVs isolated from human synovial fibroblasts and pathway analysis unveils their involvement in senescence, fibrosis and ageing as well as osteoarthritis. Functionally, the authors show that senescent synovial fibroblasts can induce senescence in surrounding cells through the release of senescence associated EVs, and that treatment of OA synovial fibroblasts with senescent EVs may cause a transcriptional switch from arthritic synovial fibroblasts to senescent synovial fibroblasts that results in reduced synthesis of pro-inflammatory and extracellular matrix molecules, potentially through the downregulation of CRE and NF-κβ signalling pathways.

Exogenous miRNAs are increasingly being shown to regulate gene expression between different species (Li et al., 2021). In particular, a growing number of studies have been testing the hypothesis that humans ingest exogenous miRNAs through the diet and that, following absorption, these miRNAs can affect human gene expression. Here, Roglia et al. report a novel example of such cross-kingdom interactions, involving *Moringa oleifera*, a plant native to tropical forests of India and food additive, and two mammal species. Building on the knowledge that *Moringa oleifera* possesses lipid modulatory properties (Barbagallo et al., 2016; Ezzat et al., 2020), the authors study the effects of *Moringa oleifera* miRNAs on lipid metabolism. Both treatment of a human liver cancer cell line (HepG2) with Moringa oleifera seed extract, as well as transfection of these cells with purified miRNAs appear to decrease intracellular lipid accumulation and induce apoptosis. Furthermore, *in vivo* administration of *Moringa oleifera* miRNAs in pre-obese mice prevent dysregulation of lipid metabolism. Further research to confirm that the miRNAs are the bioactive components of the *Moringa oleifera* extract is imperative if a new therapeutic approach based on plant miRNAs for the treatment of diseases such as obesity is to be considered.

The miRNA machinery is also involved in neurodegenerative diseases, for example, in DM1. DM1 is caused by pathogenic expansion of a CTG repeat in the DMPK gene. The expanded CUG repeat transcripts aberrantly recruit RNA-binding proteins culminating in cellular toxicity. These include proteins of the miRNA machinery, recruitment of which results in generation of aberrant small CUG RNAs. These small siRNA-like CUG-RNA molecules then can target CAG/CUG-containing RNAs. Here, Braun et al. studied transcriptome changes in DM1. They found characteristic changes in gene expression with genes bearing CTG/CAG-repeats being differentially expressed between DM1 patients and controls. Furthermore, the pathophysiology of the disease is reflected and can be predicted by the differential gene expression across patients.

Besides the above-mentioned original articles, the research topic includes a review by Nabariya et al. that further elaborates on RNA dysregulation in CXG repeat expansion diseases. In greater detail, this review summarizes the role of intracellular RNA granules and extracellular vesicles such as exosomes in neurodegeneration. Intracellular RNA granules are important to fine-tune RNA localization, stability and translation and their aberrant regulation is connected to CXG repeat expansion diseases. RNA can further be transported between cells *via* exosomes, which can transfer pathogenic molecules of CXG repeat expansion diseases from cell to cell, thereby driving disease progression. The review further summarizes recent studies showing that cytosolic RNA granules and exosomes share common content. Therefore, intracellular RNA granules and exosome loading may be related.

The research topic also contains information on different methods to study RNA interactions. Desideri et al. describe recent advances in endogenous RNA pull-down. While RNA pull-down is broadly used to study RNA-protein or RNA-RNA interactions in *in vitro* set-ups with *in vitro* transcribed transcripts as a bait, endogenous RNA pull-down assays are more relevant to identify intracellular binding partners of an RNA. Desideri et al. apply an endogenous RNA pull-down to a neuron-specific long non-coding RNA and show that both the yield of RNA-recovery and the specificity of detection of known interaction partners dramatically increase in the presence of Dextran Sulfate Sodium (DSS) salt. This modified experimental set-up may be valuable to study other RNA-RNA or RNA-protein interactions of biological significance. Furthermore, the review by Nabariya et al. provides a summary of established protocols for the isolation and characterization of exosomes and intracellular RNA granules.

We hope that further research topics will continue to celebrate the contributions of female researchers in the field of RNA research, promote their visibility and, in turn, help retain more women scientists and gender minorities in academia.
